# Skin and Soft Tissue Infections and Associated Complications among Commercially Insured Patients Aged 0–64 Years with and without Diabetes in the U.S.

**DOI:** 10.1371/journal.pone.0060057

**Published:** 2013-04-10

**Authors:** Jose A. Suaya, Debra F. Eisenberg, Christy Fang, Loren G. Miller

**Affiliations:** 1 GlaxoSmithKlineVaccines, Philadelphia, Pennsylvania, United States of America; 2 HealthCore, Inc., Wilmington, Delaware, United States of America; 3 Los Angeles BioMedical Research Center at Harbor-UCLA, Division of Infectious Diseases, Harbor-UCLA Medical Center, Torrance, California, United States of America; 4 David Geffen School of Medicine at UCLA, Los Angeles, California, United States of America; UW Medical School, United States of America

## Abstract

**Introduction:**

Skin and soft tissue infections (SSTIs) are common infections occurring in ambulatory and inpatient settings. The extent of complications associated with these infections by diabetes status is not well established.

**Methods:**

Using a very large repository database, we examined medical and pharmacy claims of individuals aged 0–64 between 2005 and 2010 enrolled in U.S. health plans. Diabetes, SSTIs, and SSTI-associated complications were identified by ICD-9 codes. SSTIs were stratified by clinical category and setting of initial diagnosis.

**Results:**

We identified 2,227,401 SSTI episodes, 10% of which occurred in diabetic individuals. Most SSTIs were initially diagnosed in ambulatory settings independent from diabetes status. Abscess/cellulitis was the more common SSTI group in diabetic and non-diabetic individuals (66% and 59%, respectively). There were differences in the frequencies of SSTI categories between diabetic and non-diabetic individuals (p<0.01). Among SSTIs diagnosed in ambulatory settings, the SSTI-associated complication rate was over five times higher in people with diabetes than in people without diabetes (4.9% vs. 0.8%, p<0.01) and SSTI-associated hospitalizations were 4.9% and 1.1% in patients with and without diabetes, respectively. Among SSTIs diagnosed in the inpatient setting, bacteremia/endocarditis/septicemia/sepsis was the most common associated complication occurring in 25% and 16% of SSTIs in patients with and without diabetes, respectively (p<0.01).

**Conclusions:**

Among persons with SSTIs, we found SSTI-associated complications were five times higher and SSTI-associated hospitalizations were four times higher, in patients with diabetes compared to those without diabetes. SSTI prevention efforts in individuals with diabetes may have significant impact on morbidity and healthcare resource utilization.

## Introduction

Skin and soft-tissue infections (SSTIs) are common reasons to seek medical care in the inpatient and outpatient settings. There were an estimated 869,800 hospital admissions in the United States (U.S.) for SSTIs in 2004.[1] Several investigations have noted that the incidence of SSTIs is increasing in both the inpatient and outpatient settings in the U.S. during the first decade of the 21^st^ Century, with increases of 29% in the inpatient setting over a 4-year period [1] and 50% in the outpatient setting over an 8-year period [2]. These SSTI incidence increases are typically attributed to the rise of community-associated methicillin resistant *Staphylococcus aureus* (CA-MRSA) as an emerging cause of SSTIs [1]–[3].

There are data suggesting patients with diabetes are at increased risk for skin infection [4]. Although one investigation reported no association between diabetes and lower limb cellulites [5], another found that individuals with diabetes were more than one and a half times more likely to develop cellulitis compared with those without diabetes [6]. In another investigation, those with type 1 and type 2 diabetes were 1.6 and 1.3 times more likely to develop an SSTI, respectively, than non-diabetic individuals [7]. Further, a Dutch investigation of more than 7,000 individuals with diabetes and 18,000 without diabetes reported that bacterial skin and mucous membrane infections were more common in those with diabetes [7]. Other studies have evaluated the incidence and severity of SSTI in people with diabetes caused by specific pathogens; however, a comparator group was not included in those investigations [5], [8]–[10].

SSTIs can be associated with serious complications such as gangrene, osteomyelitis, bacteremia, and sepsis [11]–[14]. No study, to our knowledge, has compared SSTI complication rates between individuals with and without diabetes. To address this issue, we sought to estimate rates of SSTI-associated complications in a very large cohort of patients with and without diabetes.

## Methods

### Ethics statement

All data were handled in compliance with the Health Insurance Portability and Accountability Act (HIPAA) of 1996, and a limited dataset was used for the analyses. The limited dataset contained the fields of interest specific to the study, including eligibility, medical, and pharmacy information for the specified cohort during the study period, and limited the amount of protected health information (PHI) in the dataset. The study was approved by the institutional review board at the Los Angeles BioMedical Research Institute at Harbor-UCLA Medical Center.

### Study design and data source

We performed an observational, retrospective cohort study utilizing administrative claims data consolidated in the HealthCore Integrated Research Database (HIRD). The HIRD consists of monthly eligibility status and medical and pharmacy claims from 14 health plans in the U.S. These plans include health maintenance, point-of-service, preferred provider organizations, and indemnity plans located across the Northeastern, Southeastern, mid-Atlantic, Midwestern, and Western regions. The HIRD has been used previously in multiple incidence cost studies [15]–[18].

### Study population and unit of analysis

We identified individuals aged 0–64 years in the HIRD database with at least one claim for SSTI during the period 01/01/2005 through 12/31/2010. During that study period, there were approximately 35 million lives documented in the HIRD database. We excluded individuals aged 65 and older due to limited data capture of persons of this age within commercial insurance plans because they usually have Medicare coverage. No exclusion criteria were applied to the cohort and all patients had at least one day of eligibility prior to the first SSTI episode. Only individuals with at least one SSTI claim remained in the database to be used for study analyses.

### Skin and soft-tissue infections (SSTIs)

We identified SSTIs by any of their specific International Classification of Diseases, Ninth Revision, Clinical Modification [ICD-9] clinical codes among any of the diagnoses reported in medical claims from ambulatory, emergency room, or inpatient settings for services that occurred during the study period. An SSTI episode was defined by at least one medical claim of an ICD-9 code outlined in Table 1. These codes are consistent with previous investigations using administrative data to identify SSTIs [1], [3], [7].

**Table 1:** Skin and soft tissue infections (SSTI) by group, clinical condition, and ICD-9 code.

SSTI Groups	ICD-9 Codes
Abscess/Cellulitis^a^	681.x-682.x, 035.x
Decubitus Ulcer^b^	707x
Surgical site infection & Device or Graft	
Surgical site infection	998.5x, 999.3x
Non-healing surgical wound^b^	998.83
Infection due to device or graft	996.6x
Folliculitis, Impetigo, Furuncle, Mastitis & Other SSTIs	
Folliculitis	704.8x
Impetigo	684.x
Carbuncle and furuncle	680.x
Mastitis	611.0x, 771.5x
“Other” skin and subcutaneous tissue infections	686.x

a This group also includes Erysipelas (ICD-9 code: 035.x).

b In this study, decubitus ulcer and non-healing surgical wound is considered as having an active infection if: a) clinical diagnosis is associated with prescription of any of the following antibiotics: dicloxacillin, vancomycin, cefalexin, cefazolin, linezolid, daptomycin, clindamycin, trimethoprim-sulfamethoxazole, doxycycline, minocycline, quinupristin-dalfopristin within ±10 days of the index diagnosis; and b) if there is no other concomitant clinical diagnosed infection.

The date of the earliest claim with an SSTI diagnosis was defined as the index date of the SSTI. Any two claims for SSTIs or associated complications within 30 days of each other were considered part of the same SSTI episode. After an index infection episode, any subsequent SSTI episode had to be preceded by at least 30 days without an SSTI claim to qualify as a distinct episode.

### Diabetes status

Diabetes was defined as at least one medical claim with the ICD-9 code for diabetes [250.xx] during the study period. For the purpose of this investigation we did not distinguish type 1 diabetes from type 2 diabetes due to limited accuracy in administrative data. An identified SSTI infection was attributed to a diabetic patient only if the diabetes was diagnosed before or at the time of the first SSTI episode claim.

### Classification of SSTI episodes

We categorized SSTI episodes three different ways: (1) clinical SSTI category (e.g., cellulitis, impetigo); (2) ambulatory versus inpatient onset; and (3) presence or absence of SSTI-associated complication during the episode.

### Clinical SSTI categories

Based on the ICD-9 codes for SSTI (Table 1), we categorized SSTIs into four mutually exclusive clinical groups of increasing severity based on these authors' clinical judgment. These categories of SSTIs (starting with the least severe) are as follows: (1) folliculitis, impetigo, furuncle, mastitis, and other SSTIs; (2) abscess/cellulitis; (3) decubitus ulcer; and (4) surgical site infection, device or graft, and non-healing surgical wound. If an SSTI episode had claims with multiple SSTI clinical categories, as defined above, the most severe diagnosis was used to define the episode. Because decubitus ulcers and non-healing surgical wounds may not be infected, we only considered these diagnoses as representing an SSTI if the clinical diagnosis was associated with ≥1 prescription within 10 days of the diagnosis of any of the following antibiotics: dicloxacillin, vancomycin, cefalexin, cefazolin, linezolid, daptomycin, clindamycin, trimethoprim-sulfamethoxazole, doxycycline, minocycline, or quinupristin-dalfopristin.

### SSTI by place of service of initial diagnosis

We used the place of service on the ICD-9 code claim for the first SSTI claim to classify each SSTI episode as occurring either in the ambulatory or inpatient setting. In addition, for ambulatory-onset SSTI infections, we calculated the proportion of episodes that resulted in an SSTI-related hospitalization. An SSTI-related hospitalization was defined as such if it met three conditions: (1) it was preceded by an ambulatory-onset SSTI diagnosis; (2) it contained any clinical diagnosis of an SSTI or an associated complication as one of the hospital discharge diagnoses; and (3) it occurred within the infection episode time frame. Similarly, for inpatient-onset SSTIs, we calculated re-hospitalizations as the proportion of infections resulting in an SSTI-related subsequent hospital admission. A re-hospitalization was defined as such if the SSTI, or an associated complication, was contained in any of the clinical diagnoses of the hospital discharge and if the hospitalization was within the episode infection timeframe.

### SSTI-associated complications

We also identified complications, or sequelae, typically associated with SSTIs. These complications included lymphadenitis, myositis/necrotizing fasciitis, gangrene, osteomyelitis, bacteremia, endocarditis, septicemia, or sepsis. Due to overlapping clinical spectrum, the latter four were combined into one complication group. An SSTI episode was considered to have an SSTI-associated complication if the ICD-9-based complication diagnosis occurred during the index infection timeframe. ICD-9 codes for these complications are outlined in Table 1. In our analysis, an SSTI episode could have had multiple associated complications, as complications are not mutually exclusive. Based on clinical experience, we considered osteomyelitis to be associated with an SSTI episode even if it occurred up to 90 days from the beginning of the SSTI infection (index date).

### Statistical Analyses

The unit of analysis was a SSTI episode. We evaluated overall SSTI-associated complication and hospitalization rates within the infection timeframe associated with each SSTI group by place of service of initial diagnosis stratified by diabetes status. We then estimated specific SSTI-associated complication rates for each SSTI category. We used the two-proportion z-test to obtain p-values. All analyses were performed using SAS® 9.2 (SAS Institute Inc, Cary, NC) and an alpha level of significance of 0.05 was used.

## Results

### SSTIs by diabetes status and age group

During the study period of 2005 to 2010, we identified 140,652 diabetic patients and 1,539,692 non-diabetic patients aged 0–64 years with at least one SSTI episode. There were 2,227,401 SSTI episodes, 10.0% of which occurred in patients with diabetes (Table 2). There were differences in the age group distribution of SSTIs between patients with and without diabetes (p<0.01). About three quarters (75.6%) of all SSTIs in patients with diabetes were in those aged 45–64 years. Most of the remaining SSTIs (22.8%) in diabetic patients occurred in individuals aged 18–44 years. In contrast, many (43.2%) of the SSTIs in patients without diabetes occurred in individuals aged 18–44 years. The remaining 25.5% and 31.3% of the SSTIs were in individuals aged 0–17 years and 45–64 years, respectively. Fewer SSTI episodes were initially diagnosed in ambulatory settings in patient with diabetes (88.7%) than in those without diabetes (96.2%, p<0.01). This association was similar across all age groups.

**Table 2:** Distribution of skin and soft tissue infection by diabetes status and age group from 2005 through 2010.

	SSTI Episodes*					
	In Diabetic Individuals			In Non-diabetic Individuals		
Age group	All SSTIs	As % of all	% of SSTIs in age group diagnosed in ambulatory settings	All SSTIs	As % of all	% of SSTIs in age group diagnosed in ambulatory settings
0–17 years	3,602	1.6%	94.1%	512,420	25.5%	98.0%
18–44 years	50,497	22.8%	90.2%	866,098	43.2%	96.7%
45–64 years	167,546	75.6%	88.1%	627,238	31.3%	94.1%
0–64 years	221,645	100.0%	88.7%	2,005,756	100.0%	96.2%

* Difference in frequency distribution of SSTIs by age group between individuals with and without diabetes was statistically significant (p<.01).

### SSTI categories by place of service of initial diagnosis

There were differences in the frequency distribution of group of SSTIs between patients with and without diabetes (p<0.01) within place of initial diagnosis. Among SSTIs initially diagnosed in ambulatory settings, abscess and cellulitis represented 65.6% and 59.3% of all the SSTI episodes in patients with and without diabetes, respectively (Table 3). The SSTI category of folliculitis, impetigo, furuncle, mastitis, and other SSTIs was the second largest group of SSTIs in both patients with and without diabetes (22.6% vs. 37.8%). About 8.1% and 0.9% of SSTIs episodes in patients with and without diabetes were decubitus ulcers.

**Table 3:** Skin and soft tissue infection-associated complication and hospitalization rates by diabetes status, major initial clinical diagnosis, and setting from 2005 through 2010.

	SSTI Episodes^a^				Any SSTI-associated Complication					SSTI-associated Subsequent Hospitalization^b^				
	In Diabetic Individuals		In Non-diabetic Individuals		In Diabetic Individuals		In Non-diabetic Individuals		Differ-ence	In Diabetic Individuals		In Non-diabetic Individuals		Differ-ence
Initial diagnosis setting and group of SSTIs	N	As a % by setting	N	As a % by setting	N	%	N	%	p-value	N	%	N	%	p-value
**Initially Diagnosed in Ambulatory Settings**														
Abscess/Cellulitis	129,006	65.6	1,144,576	59.3	3,048	2.36	9,047	0.79	<.01	4,310	3.34	14,377	1.21	<.01
Decubitus Ulcer	15,976	8.1	17,663	0.9	5,175	32.39	2,357	13.34	<.01	3,697	23.14	1,825	8.64	<.01
Surgical Site Infection & Device or Graft	7,097	3.6	37,334	1.9	736	10.37	1,861	4.98	<.01	867	12.22	2,475	6.26	<.01
Folliculitis, Impetigo, Furuncle, Mastitis & Other SSTIs	44,494	22.6	730,194	37.8	702	1.58	3,138	0.43	<.01	736	1.65	2,796	0.37	<.01
**All SSTIs in subgroup**	196,573	100.0	1,929,767	100.0	9,661	4.91	16,403	0.85	<.01	9,610	4.89	21,473	1.05	<.01
**Initially Diagnosed in Inpatient Settings**														
Abscess/Cellulitis	12,656	50.5	41,389	54.5	3,335	26.35	6,297	15.21	<.01	1,380	10.90	2,458	5.52	<.01
Decubitus Ulcer	3,802	15.2	2,403	3.2	2,397	63.05	1,236	51.44	<.01	1,059	27.85	566	22.39	<.01
Surgical Site Infection & Device or Graft	7,438	29.7	26,939	35.5	3,159	42.47	8,225	30.53	<.01	1,200	16.13	2,937	10.31	<.01
Folliculitis, Impetigo, Furuncle, Mastitis & Other SSTIs	1,176	4.7	5,258	6.9	310	26.36	754	14.34	<.01	146	12.41	421	7.59	<.01
**All SSTIs in subgroup**	25,072	100.0	75,989	100.0	9,201	36.70	16,512	21.73	<.01	3,785	15.10	6,382	7.84	<.01

a Difference in the frequency distribution of SSTI clinical groups between patients with and without diabetes was statistically significant (p<.01) in both those diagnosed in ambulatory settings and those diagnosed in inpatient settings.

b For patients with an initial diagnosis in the inpatient setting, this counts as a re-hospitalization.

Similar to the ambulatory setting, among SSTIs initially diagnosed in inpatient settings, abscess and cellulitis was the most common SSTI category, accounting for about 50.5% and 54.5% of SSTIs in patients with and without diabetes, respectively (Table 3). However, the group of surgical site, device or graft infections was more common in inpatient settings than outpatient settings, representing 29.7% and 35.5% of SSTIs in patients with and without diabetes, respectively. Decubitus ulcers represented 15.2% and 3.2% of the SSTIs initially diagnosed in inpatient settings in patients with and without diabetes, respectively.

### SSTI-associated complications

For each SSTI category, higher complication rates were observed in SSTIs occurring in patients with diabetes than without diabetes (Table 3). Among SSTIs initially diagnosed in ambulatory settings, the SSTI-associated complication rate was more than five times higher in patients with diabetes than without diabetes (4.9% vs. 0.8%, p<0.01). Similarly, SSTI-associated complication rates among SSTIs initially diagnosed in inpatient settings were over one and half times higher in diabetic than in non-diabetic individuals (36.7% vs. 21.7%, p<0.01). SSTI-associated complication rates were highest in decubitus ulcers and the combined category of surgical site infections/device or graft infection. For example, 32% and 13% of the decubitus ulcers initially diagnosed in an ambulatory setting were associated with complications in patients with and without diabetes, respectively. SSTI-associated complication rates were the lowest in the clinical group of folliculitis, impetigo, furuncle, mastitis, and other SSTIs, observed in 2.4% and 0.8% of the episodes in patients with and without diabetes, respectively.

### SSTI-related hospitalizations

Among SSTIs diagnosed in the ambulatory care setting, SSTI-associated hospitalizations were 4.9% in patients with diabetes and 1.1% in non-diabetic patients, respectively (Table 3). Among SSTIs diagnosed in the inpatient care setting, SSTI-associated subsequent (re)hospitalizations were 15.1% and 7.8% in patients with and without diabetes, respectively (Table 3). For SSTIs diagnosed in the ambulatory setting, SSTI-related hospitalizations rates varied by the SSTI category, ranging from 0.4% in the folliculitis group in non-diabetic individuals to 23.1% in decubitus ulcers in diabetic individuals. For SSTIs diagnosed in inpatient setting, SSTI-related subsequent hospitalizations also varied by the SSTI clinical category, ranging from 5.5% in the abscess/cellulitis group in non-diabetic patients to 27.9% in decubitus ulcers in diabetic patients. Again, for each group (except the folliculitis group), SSTIs in patients with diabetes were associated with significantly higher subsequent hospitalization rates than those patients without diabetes.

### SSTI-specific complications

Specific SSTI-associated complications are outlined in Tables 4 and 5. Among patients with SSTIs initially diagnosed in ambulatory settings, osteomyelitis was the most commonly associated complication, occurring in 3.3% and 0.4% of patients with and without diabetes, respectively (p<0.01 for difference in proportions). Among SSTIs initially diagnosed in inpatient settings, bacteremia/endocarditis/septicemia/sepsis was the most common SSTI-associated complication occurring in 25% and 16% of patients with and without diabetes, respectively (p<0.01 for difference of proportions).

**Table 4:** Complications of Lymphadenitis and Myositis/Necrotizing Fasciitis by diabetes status, major initial clinical diagnosis, and setting from 2005 through 2010.

				Type of SSTI-associated Complication^a^					
	SSTI Episodes			Lymphadenitis			Myositis/Necrotizing Fasciitis		
	In Diabetic Individuals		In Non-diabetic Individuals	In Diabetic Individuals	In Non-diabetic Individuals	Differ-ence	In Diabetic Individuals	In Non-diabetic Individuals	Differ-ence
Initial Diagnosis Setting and group of SSTIs	N		N	N (%)	N (%)	p-value	N (%)	N (%)	p-value
**Initially Diagnosed in Ambulatory Settings**									
Abscess/Cellulitis	129,006	1,144,576		110 (0.09%)	1,605 (0.14%)	<.01	106 (0.08%)	279 (0.02%)	<.01
Decubitus Ulcer	15,976	17,663		12 (0.08%)	10 (0.06%)	.26	78 (0.49%)	63 (0.36%)	.03
Surgical site infection & Device or Graft	7,097	37,334		3 (0.04%)	29 (0.08%)	.11	10 (0.14%)	24 (0.06%)	.05
Folliculitis, Impetigo, Furuncle, Mastitis & Other SSTIs	44,494	730,194		39 (0.09%)	692 (0.09%)	.31	27 (0.06%)	79 (0.01%)	<.01
**All SSTIs in subgroup**	196,573	1,929,767		164 (0.08%)	2,336 (0.12%)	<.01	221 (0.11%)	445 (0.02%)	<.01
**Initially Diagnosed in Inpatient Setting**									
Abscess/Cellulitis	12,656	41,389		34 (0.27%)	559 (1.35%)	<.01	294 (2.32%)	505 (1.22%)	<.01
Decubitus Ulcer	3,802	2,403		4 (0.11%)	3 (0.12%)	.41	88 (2.31%)	31 (1.29%)	<.01
Surgical site infection & Device or Graft	7,438	26,939		2 (0.03%)	31 (0.12%)	<.01	49 (0.66%)	106 (0.39%)	<.01
Folliculitis, Impetigo, Furuncle, Mastitis & Other SSTIs	1,176	5,258		1 (0.09%)	9 (0.17%)	.20	17 (1.45%)	40 (0.76%)	.03
**All SSTIs in subgroup**	25,072	75,989		41 (0.16%)	602 (0.79%)	<.01	431 (1.72%)	682 (0.90%)	<.01

a Complications are not mutually exclusive.

**Table 5:** Complications of gangrene, osteomyelitis, and bacteremia/endocarditis/septicemia/sepsis by diabetes status, major initial clinical diagnosis, and setting from 2005 through 2010.

	Type of SSTI-associated complication^a^								
	Gangrene			Osteomyelitis			Bacteremia/Endocarditis/Septicemia/Sepsis		
	In Diabetic Individuals	In Non-diabetic Individuals	Differ-ence	In Diabetic Individuals	In Non-diabetic Individuals	Differ-ence	In Diabetic Individuals	In Non-diabetic Individuals	Differ-ence
Initial Diagnosis Setting and group of SSTIs	N (%)	N (%)	p-value	N (%)	N (%)	p-value	N (%)	N (%)	p-value
**Initially Diagnosed in Ambulatory Settings**									
Abscess/Cellulitis	551 (0.43%)	432 (0.04%)	<.01	1,783 (1.38%)	3,995 (0.35%)	<.01	1,050 (0.81%)	3,161 (0.28%)	<.01
Decubitus Ulcer	1,239 (7.76%)	372 (2.11%)	<.01	4,014 (25.13%)	1,522 (8.62%)	<.01	1,273 (7.97%)	735 (4.16%)	<.01
Surgical site infection & Device or Graft	33 (0.46%)	52 (0.14%)	<.01	241 (3.40%)	972 (2.60%)	<.01	525 (7.40%)	909 (2.43%)	<.01
Folliculitis, Impetigo, Furuncle, Mastitis & Other SSTIs	113 (0.25%)	159 (0.02%)	<.01	344 (0.77%)	1,005 (0.14%)	<.01	269 (0.60%)	1,309 (0.18%)	<.01
**All SSTIs in subgroup**	1,936 (0.98%)	1,015 (0.05%)	<.01	6,382 (3.25%)	7,494 (0.39%)	<.01	3,117 (1.59%)	6,114 (0.32%)	<.01
**Initially Diagnosed in Inpatient Setting**									
Abscess/Cellulitis	669 (5.29%)	358 (0.86%)	<.01	1,183 (9.35%)	1,535 (3.71%)	<.01	2,139 (16.90%)	4,244 (10.25%)	<.01
Decubitus Ulcer	803 (21.12%)	172 (7.16%)	<.01	1,429 (37.59%)	393 (16.35%)	<.01	1,116 (29.35%)	861 (35.83%)	<.01
Surgical site infection & Device or Graft	123 (1.65%)	137 (0.51%)	<.01	598 (8.04%)	1,812 (6.73%)	<.01	2,747 (36.93%)	6,752 (25.06%)	<.01
Folliculitis, Impetigo, Furuncle, Mastitis & Other SSTIs	76 (6.46%)	62 (1.18%)	<.01	115 (9.78%)	217 (4.13%)	<.01	189 (16.07%)	510 (9.70%)	<.01
**All SSTIs in subgroup**	1,671 (6.66%)	729 (0.96%)	<.01	3,325 (13.26%)	3,957 (5.21%)	<.01	6,191 (24.69%)	12,367 (16.27%)	<.01

a Complications are not mutually exclusive.

## Conclusions

In our investigation of more than 2 million SSTIs from people with and without diabetes in a large database of commercially insured persons in the U.S., we found several novel observations about SSTIs. First, the majority of SSTIs were diagnosed in ambulatory settings for patients with and without diabetes, but significantly more infections were diagnosed in inpatient settings for patients with diabetes compared to patients without diabetes. Second, in both inpatient and ambulatory settings, for both patient groups, abscess and cellulitis were the most commonly diagnosed infections, although these were more common among those with diabetes compared with people without diabetes. Third, SSTI-associated complications were five-fold and one and a half-fold more common in individuals with diabetes than without diabetes for SSTIs initially diagnosed in ambulatory and inpatient setting, respectively. Finally, SSTI-related subsequent hospitalizations were more common in patients with diabetes in both the inpatient and outpatient settings.

There are many reasons why persons with diabetes are felt to be at increased risk for bacterial infections, such as SSTIs. These include impaired leukocyte chemotaxis, adherence, and intracellular bacterial killing [19]. Additionally, antigen-specific cell-mediated immunity and impaired proliferative responses to certain pathogens, such as *S. aureus*, the most commonly recognized cause of skin infection, has been described [19]. Despite descriptions of impaired immunity, clinical data supporting the association between diabetes and increased infection are very limited. While there are data suggesting that diabetic individuals are associated with higher risk of SSTIs [6], [7], this is the first study, to our knowledge, to investigate the morbidity associated with SSTIs, and evaluate the differences in type and complications between patients with and without diabetes.

Shah, et al. found that individuals with diabetes have a 1.21 increased risk ratio (RR) for all infectious diseases compared with individuals without diabetes [6], They also observed that diabetic individuals were at higher risk for specific infectious diseases, for example, upper respiratory tract infections (RR 1.18), pneumonia (RR 1.46), and cystitis (RR 1.39). They did examine the risk of one skin infection, cellulitis, and found an increased risk in those with diabetes (RR 1.81) and also found an increased risk of osteomyelitis (RR 4.39). However, they did not examine other categories of skin infections, SSTI-associated hospitalizations, or SSTI-associated complications. Muller et al. also found type 1 and type 2 diabetic patients were at increased risk of bacterial skin and mucous membrane infections (ORs 1.59 and 1.33, respectively) [7]. However, they did not examine specific skin infections or their complications. Study populations in both studies were extremely small compared to the population examined in the current investigation.

In our population, not surprisingly, the relatively less severe SSTIs − folliculitis, impetigo, furuncles, and mastitis − were associated with relatively low rates of associated complications (1.6% and 0.4% in patients with and without diabetes, respectively) and hospitalizations (1.7% and 0.4%, respectively). Decubitus ulcers were the SSTI category with the highest proportions of associated complications (32.4% and 13.3%) and hospitalizations (23.1% and 8.6%). Surgical site infections/device or graft also had relatively high proportions of associated complications (10.4% and 5.0%) and hospitalizations (12.2% and 6.3%). These findings are consistent with our anecdotal clinical observations that more serious SSTIs are associated with higher likelihoods of complications and subsequent hospitalization.

We found SSTI-associated complications were generally uncommon in ambulatory onset SSTIs, occurring in less than 4% of SSTIs. The most common associated complication was osteomyelitis, which occurred in 3.2% and 0.4% of SSTIs in patients with and without diabetes, respectively. All other SSTI-associated complications were relatively uncommon occurring ≤1.0% of the time. As expected, SSTI-associated complications rates in the inpatient setting were higher than in SSTIs initially diagnosed in ambulatory settings, with bacteremia/endocarditis/septicemia/sepsis group being the most common, occurring in 24.7% and 16.3% of those SSTIs in patients with and without diabetes, respectively.

Our investigation has limitations. First, our study employed administrative claims data. While cellulitis and abscesses are different clinical entities, ICD-9 codes group them together, and the accuracy of SSTI diagnosis from administrative claims data has not been fully validated. Thus, we may have overestimated or underestimated the true number and groups of SSTIs compared with medical records review. Second, our study included only persons aged 0–64, as persons over 65 years were uncommon in our database. It is likely that SSTIs and their associated complications were more common in the population aged 65 years and older [20]–[22]. A third limitation is that we do not have microbiologic correlation with SSTIs. Such data may lead to insights as to which pathogens may disproportionally affect individuals with diabetes. However, the HIRD databases do not contain inpatient microbiology data. Even if microbiologic data were available, it may be of limited utility given many SSTIs, such as cellulitis without abscess, cannot be easily cultured, or clinicians often choose not to perform bacterial cultures in persons with SSTIs [23]. Fourth, subsequent hospitalizations were assumed to be related to SSTIs, however it is possible that patients were hospitalized for other reasons and that the skin infection remained ongoing. Fifth, our study population consisted of individuals commercially insured and thus our findings may not be generalizable to uninsured or patients with Medicaid. Another limitation of our study was our inability to distinguish risk of SSTI between type 1 and type 2 diabetics. Administrative data has been found to be a poor means to distinguish these two diabetic types in children and younger adults in which both types are relatively prevalent [24]. Finally, as our unit of analysis was an SSTI episode and its complication rates, this study did not focus in estimating SSTI incidence among persons at risk stratified by diabetes status. Such an analysis would have broadened the understanding of the role of diabetes in not only the risk of complications associated with SSTIs but also in the risk of SSTIs themselves.

There are strengths to our study. First, we employed an extremely large database of the commercially insured U.S. population. Second, the population under study was geographically diverse, representing insured adults and children throughout the U.S. In addition, we employed a comprehensive approach to SSTIs, examining both ambulatory- and inpatient-onset SSTIs, whereas almost all other investigations of SSTIs have focused on one setting only. Finally, despite our lack of microbiologic information, our results are contemporary and reflect SSTIs that occur in the era of CA-MRSA, which has changed the epidemiology of skin infections in the first decade of the 21st century [25]–[26].

In summary, we found that diabetic patients with SSTIs have higher rates of both SSTI-associated complications and subsequent hospitalizations. Therefore, successful preventive interventions for SSTIs may have the greatest impact among persons with diabetes.
